# Two-Year Use of Care Robot Zora in Dutch Nursing Homes: An Evaluation Study †

**DOI:** 10.3390/healthcare7010031

**Published:** 2019-02-19

**Authors:** Chantal Huisman, Helianthe Kort

**Affiliations:** 1 Research Center Sustainable and Healthy Living Utrecht, Research Group Technology for Healthcare Innovations, Utrecht University of Applied Sciences, 3584 CJ Utrecht, The Netherlands; helianthe.kort@hu.nl; 2 Department of the Built Environment, Chair Building Healthy for Environments Future Users, Eindhoven University of Technology, 5612 AZ Eindhoven, The Netherlands

**Keywords:** long-term care facilities, older adults, gerontechnology

## Abstract

The use of the Zora robot was monitored and evaluated in 14 nursing care organizations (15 locations). The Zora robot, a Não robot with software, is designed as a social robot and used for pleasure and entertainment or to stimulate the physical activities of clients in residential care. In the first year, the aim was to monitor and evaluate how the care robot is used in daily practice. In the second year, the focus was on evaluating whether the use of Zora by care professionals can be extended to more groups and other type of clients. Interviews, questionnaires and observations were used as instruments to reveal the progress in the use of the robot and to reveal the facilitators and barriers. Care professionals experienced several barriers in the use of the robot (e.g., start-up time and software failures). The opportunity they had to discuss their experience during project team meetings was seen as a facilitator in the project. Furthermore, they mentioned that the Zora robot had a positive influence on clients as it created added value for the care professionals in having fun at work.

## 1. Introduction

These days, life without technology is unthinkable and more and more care organizations incorporate technology in daily care routines. Technology implemented in care organizations comprises video conferencing not only for telecare and teleconsultations but also for the use of robots. There are different types of robots in healthcare, for example service robots and social robots [1]. Service robots mostly serve as an aid for elderly or disabled people. Social robots are developed for social interaction with elderly people, for example, to improve their health and psychological well-being. Different social robots are already available on the market for the elderly [2]. Research shows that the use of social robots in the care of people with dementia has intriguing possibilities, addressing support issues in caring for people with dementia [3]. In healthcare, robots are used for rehabilitation therapy or to assist persons in their daily activities. Furthermore, robots are now also used for social activities and entertainment. One example of such a social robot is PARO. The PARO seal robot is used for the social support for older adults [4]. It is known that the PARO seal robot, when offered to persons with dementia in nursing homes, will lead to engagement. Several other studies show that the PARO robot [5,6] has a positive influence on older persons with dementia.

**Figure 1:**
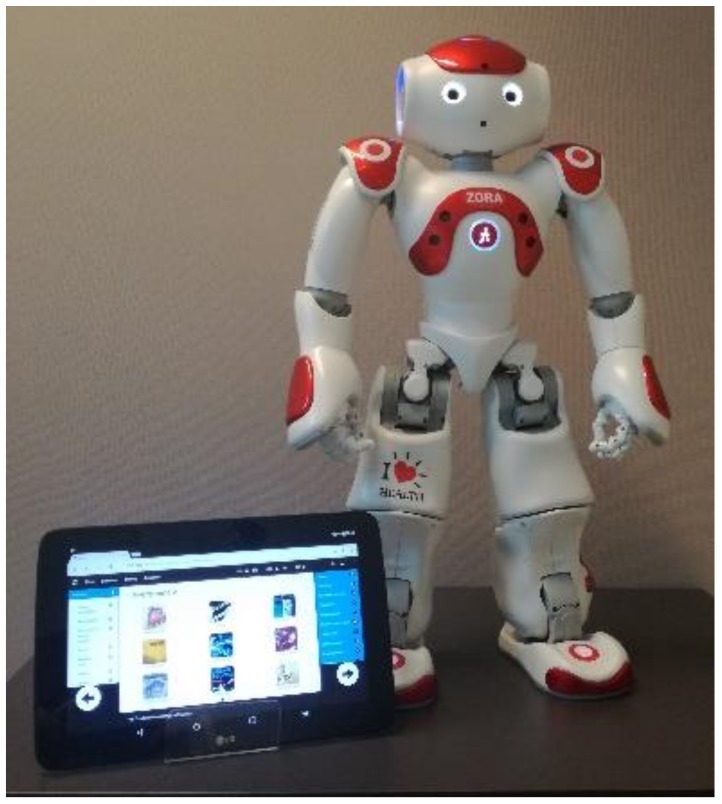
A picture of the care robot Zora with a tablet for control.

**Figure 2:**
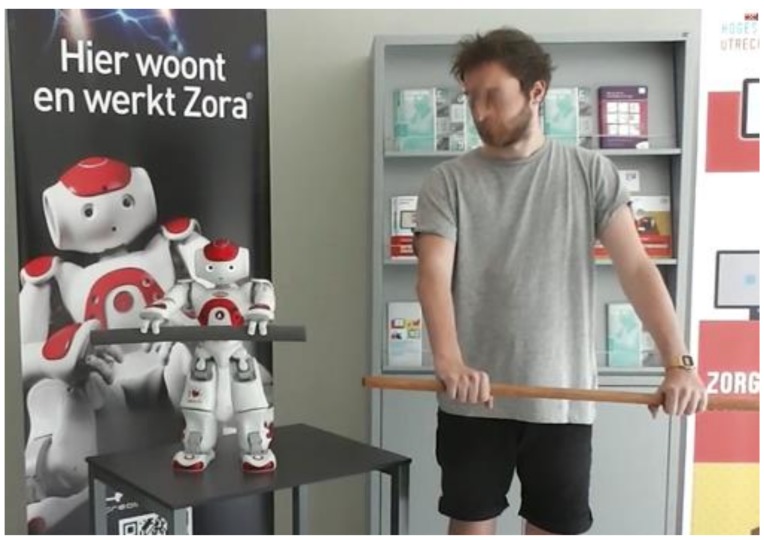
A demonstration of the robot Zora (student physiotherapy).

This paper reports on the use of the Zora robot (Figure 1) also as a social robot in nursing homes. Zora is an acronym made up of the first letters of the Dutch words for care, elderly, rehabilitation and animation. Zora is a humanoid robot of 57 cm in height, with functional sensors for seeing and hearing. Zora is a Não robot with hardware developed by Softbank Robotics and with software made by a Belgium company (Zorabots). In this paper, the robot is referred to as Zora. Zora is used for rehabilitation practice (see Figure 2), social activities and entertainment. Não robots are also used for children with autism, and they contribute to the development of those children [7]. A recent study on understanding older people’s use of technology showed that performance expectancy, effort expectancy, and perceived privacy and security are direct predictors of older people’s intentions to use technology innovations such as videoconferencing [8]. Another recent study showed that for the implementation of eHealth in homes for the elderly, the preconditions must be clear and, therefore, more qualitative research is needed to reveal the perspectives of older people on technology and to investigate their motives for considering technology [9]. In the Netherlands, the use of care robots by professionals in care for the elderly increased from 3% in 2016 to 8% in 2017 [10].

**Figure 3:**
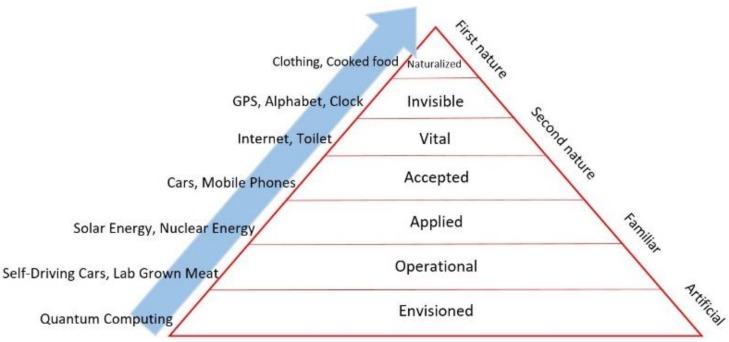
The pyramid of technology.

The technology innovation stage and the extent of take-up in society can be illustrated by the pyramid of technology which distinguishes between the technology stages of envisioned, operational, applied, accepted, vital, invisible and naturalized [11]; see also Figure 3. Envisioned means just having an idea for a technological innovation, while operational means this idea has moved into something that does work. Applied and accepted mean that the technology works in practice and is accepted by users. Vital, invisible and naturalized apply to a technology innovation which is part of daily life (mobile phone, electricity or cooking, for instance). In this pyramid of technology, the Zora robot is still in the pioneer phase, namely between the operational and applied stages.

Fourteen care organizations in the Netherlands formulated the ambition to use the social robot Zora in daily (residential) care for older persons with a long-term care demand, and therefore, the project “Care innovation with Zora” was started. The project is supported by IVVU (Institutions for Nursing and Care in Utrecht), a regional association for long-term care facilities in the Utrecht region, The Netherlands.

The project objective is to innovate nursing home care by introducing robot technology to care professionals and making them acquainted with the Zora robot up to the level of acceptance. Furthermore, the project was initiated to get a better insight into what technology can mean for daily practice and to investigate the facilitators and barriers. The main research questions were how to apply the use of the Zora robot in residential care and what is the perspective of care professionals regarding the acceptance of the robot. Therefore, this study investigated to which extent professionals and clients engage with the Zora robot and/or accept the robot. In addition, we investigated the facilitators and barriers in using the Zora robot to formulate recommendations in order to move away from the pioneer phase towards the acceptance stage of this technology innovation.

## 2. Materials and Methods

### 2.1. Design

This practice-based study used a mixed-method design. Quantitative data were collected in relation to mood and involvement with the Zora robot while qualitative data included observations and interviews. The following questions were leading:

- For which client groups is Zora used?
- Is the robot used in a group setting and/or in an individual setting?
- How many departments and/or locations of the organizations did work with Zora?
- Which type of care professionals uses Zora?

### 2.2. Locations and Process

**Figure 4:**
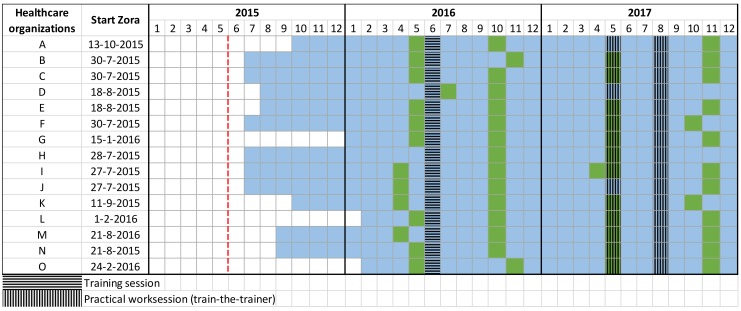
The monitoring and evaluation scheme of the project from the start in 2015. The first year of monitoring and evaluation was 2016, and 2017 was the second year. The field trial period of each location is indicated in blue. The measurement moments (observations and questionnaires) are illustrated in green. The start of the IVVU project is indicated by the red dotted line.

The use of the Zora robot was investigated on 15 locations in the Utrecht region. The project was initiated and started in 2015 by the association of care-organizations in the Utrecht region (IVVU). Students and research staff visited the care organizations in spring and autumn of 2016 and spring and late autumn in 2017. The starting times of the 14 care-organizations (15 locations) are given in Figure 4, since this varied per location. The Não robot with software from Zorabots, referred to as Zora in this paper, was purchased from Zorabots. All fifteen locations received the Zora robot and were allowed to choose to use Zora for any purpose in care. The basic functionalities of the robot include walking, sitting, talking, moving and dancing, in other words, social activities and entertainment. Care professionals could use the robot for entertainment and rehabilitation.

**Figure 5:**
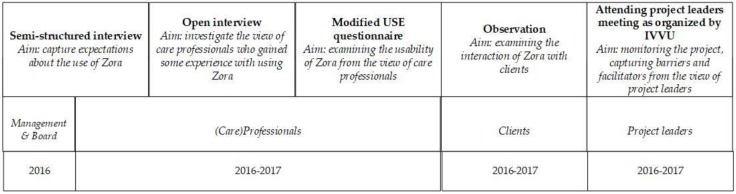
An overview of the methods used and the aims during the two-year research.

To monitor the project and to capture the facilitators and barriers of each subproject on location, the research staff attended the central meetings for project leaders organized by IVVU, which were held every six to eight weeks. The project leaders within each of the participating care organizations visited these meetings. At these meetings, notes were taken, categorized and systematized via open coding. This was done by marking the notes explaining the views and opinions of the project leaders about the use of Zora. In addition, an axial coding process was executed to rearrange the codes and to develop themes which indicate the facilitators and barriers in the use of Zora in daily practice. An overview of all the instruments used can be found in Figure 5.

#### 2.2.1. Semi-Structured Interviews

The semi-structured interviews used were to capture the expectations of board members and care professionals on the added value of Zora. These interviews were also executed to identify the facilitators and barriers prior to the use of Zora on a daily basis. The semi-structured interviews were based on the 7A theory for implementation [12]. The questions asked were about the awareness, availability, accessibility, affordability, acceptability, appropriateness and adequacy of Zora. The questions were “When and in what way did you hear about Zora?”, “What made you decide to start with the project?”, “How does the use of the robot fit with the strategy, and is it aligned with your policy for daily care and the client’s lifestyle?”, “Did you work with a business plan?”, “How do you want to create awareness about the use of Zora in care?”, “What is the expected added value of using Zora?” and “Do you also work with other organizations with respect to the use of Zora?” All the interviews were recorded after permission was given. The Management and Board (*N* = 15) and professional carers (*N* = 20) were interviewed. The analysis of the phrases in the text was carried out with the open coding method followed by axial coding. The codes used were in analogy with the topics in the 7A theory [12], and the codes used were such as awareness, use in daily practice, willingness/eagerness to use, acceptance, technical/software issues, experiences with Zora, skills and functions of Zora.

#### 2.2.2. Open Interviews

Open interviews were conducted to investigate the view of care professionals who gained some experience with using Zora about the added value of Zora and to reveal the barriers and/or facilitators for using the Zora robot in nursing homes after having gained experience with the use of Zora. To examine the actual use of Zora in practice, students visited the organizations for at least one day or up to four days to monitor how the organizations worked with Zora throughout the day. The variation in number of days was due to the availability and willingness of the project leader and the organization to cooperate. Students were only welcomed in 12 care organizations. Organizations were also given the opportunity to ask students for support with the use of Zora in daily practice; students had been trained in using the robot. Via this easy approachable contact with professionals, the facilitators and barriers could be revealed. In addition, the functionalities of how Zora could be improved from the perspective of daily practice was investigated.

#### 2.2.3. Modified Use Questionnaire

A modification of the Usefulness, Satisfaction, Ease of Use (USE) questionnaire from Lund (2001) [13] was used. The internal validity was reached via discussions with students and staff in a think-aloud session about which questions needed to be added or replaced. Adjustments were made for the context of the use of the Zora robot, and the topics that were added concerned the effects Zora might have on clients and about the staff’s work satisfaction. The questionnaire included the following dimensions: usability, ease of use, ease of learning, satisfaction, effects and work experience. Staff could also add remarks. The participants who filled out the USE questionnaire during the different research periods were all staff who worked with Zora. The participants had different ages, gender, education(al) (levels), functions and experiences with Zora (Table 1). There were participants whose position, for example, was activity counsellor, nurse, trainee, policy maker, physiotherapist or volunteer. Some of them had worked with Zora for a couple of hours; others worked with Zora on a regular basis of once a week or more. The majority of the staff received training in working with Zora. The students monitored the use of Zora in 13 organizations by using the modified USE questionnaire.

#### 2.2.4. Observations

**Figure 6:**
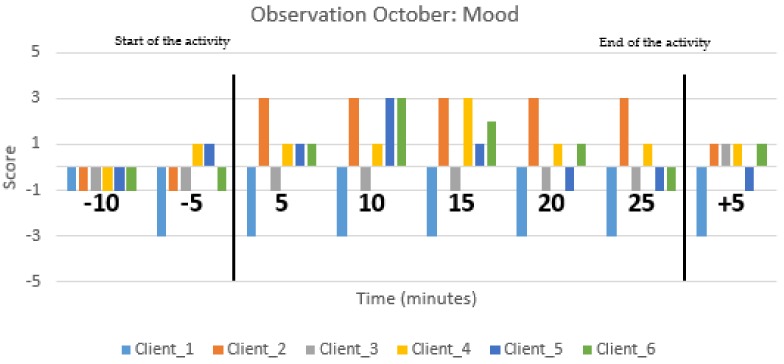
An observation score on mood during a group activity with the Zora robot in which six clients participated. All clients are from a psychogeriatric ward. Each colored bar represents one client. On the X-axis, the time prior to an activity (−10 and −5), the time during the activity (0–25) and after the activity (+5) are given. On the Y-axis, the mood score (−5 to +5) is given. The black vertical lines mark the start and end of the group activity with Zora.

**Figure 7:**
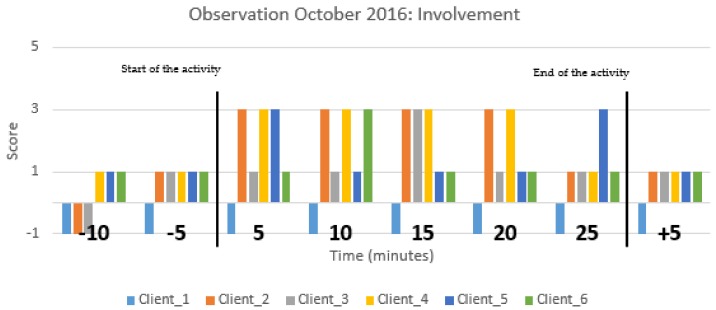
An observation score on involvement during a group activity with the Zora robot in which six clients participated. All clients are from a psychogeriatric ward. Each colored bar represents one client. On the X-axis, the time prior to an activity (−10 and −5), the time during the activity (0–25) and the time after the activity (+5) are given. On the Y-axis, the involvement score (−1 to + 5) is given. The black vertical lines mark the start and end of the group activity with Zora.

Furthermore, activities with Zora were monitored via an observation method in accordance with Groenewoud et al. (2017) [14]. The validation of this method was set within their project. Students conducted more than 150 observation sessions during the development of games for people with dementia. With this method, the interaction of older people with a technology innovation was rated on mood and involvement. In each care organization, at least one group activity with Zora was observed. The observations were executed by students who had been instructed on how to conduct the observations. Students were trained to keep a neutral attitude towards clients and staff and also taught how to work in accordance with the protocol and how to use the observation form (see Supplement A). Two students were assigned to each observation, so both could observe half of the group. The group’s activities included the participation of six to ten clients. During the observations, clients’ moods and their involvement with Zora were scored from 15 min before the start of the activity, during the activity and at least 15 min after the activity with the Zora robot. Each student observed a maximum of four clients. All clients were older adults with a high intense care demand, similar to people with psychogeriatric problems. The scale scores mood range from −5 to +5 for very negative emotions (e.g., sad, afraid) to happy and joyful (e.g., laughing/relaxed facial expression and posture) respectively. The scale for involvement is from −1, scored for turning inwards (e.g., eyes closed or looking at the ground), to +5, scored for highly involved (e.g., concentrated on the activity, no distractions) [14]. The average scores were used to calculate the extent of mood and involvement of an individual client. Activities with Zora were conducted by care professionals and/or occasionally by volunteers or trainees. In addition, clients and professionals were questioned about their experience with Zora. Questions such as “Would you like to do the activity again?” and “What did you dislike about the activity with Zora?” were asked. An example of the observation on mood is given in Figure 6, and one for involvement is given in Figure 7 (both with 6 clients). The Y-axis gives the average score per client during one session on mood (Figure 6) or involvement (Figure 7) for each client. The X-axis reflects the time of the observation given in blocks of 5 min, starting, prior, during and after the activity with Zora. Each colored bar reflects one individual (client).

Ethical considerations: Ethical approval was given via the client and employees councils. All participating care organizations informed their employees and clients councils, and all received approval from their client councils to execute the project. The topic is not covered for review by the Medical Research Involving Human Subjects Act (http://www.ccmo-online.nl). Data from clients and professionals are protected in the cloud of the university only and are anonymized. Clients and professionals participated on a voluntary basis and were free to stop with the project/activity at any moment.

## 3. Results

The views of the Management and Board, followed by the views of the professional carers and the findings of the client observations are presented at the end of this section.

### 3.1. Management and Board

In total, fourteen board members and one member from the management staff were interviewed about their expectations regarding Zora.

Most of the board members and management staff first heard about Zora through the IVVU.

The view of the Management and Board is that participating in this project pioneer phase is aligned with the mission and vision of the care organizations. A quote given by one of the board members is “Zora fits in with the vision of the organization, in which we consider the well-being of our clients.”

Furthermore, board members felt that the project offered the possibility to gain experience with technology in care. One of the quotes is “We want to anticipate the future.”

Board members think that staff could explore and gain more experience in understanding the use of robots in daily care practice. A quote: “We want our employees to be exposed to technology innovations in healthcare.”

They also envisioned that the project will enhance the view of staff on technological innovations in a positive way and stimulate the curiosity of professionals. 

During the project, it became clear that it is necessary to improve the ICT (Information and Communications Technology)infrastructure in the care organization in order to contribute to the optimal functioning of Zora. That is why in the short-term Wi-Fi access in the buildings was improved. In addition, an implementation for further improvements of the ICT infrastructure was placed on the agenda.

Board members also expected that a snowball effect will occur in sharing knowledge between professionals about using social robots in general and more specifically about hints and tips for using Zora. 

The expectation is that in the long run, working with Zora will be part of the daily routine.

At the start of the project, the care organizations did not collaborate with other organizations which are not part of the IVVU project. A quote: “There is no collaboration at this moment, but in the future, this would be possible when the use of Zora increases.”

### 3.2. Professional Carers

#### 3.2.1. Professional Carers’ Views on Using Zora in Care

Based on the work satisfaction questions in the modified USE questionnaire, seventeen professionals working on the wards gave their views on how they experienced working with Zora. A third of the professionals stated that there is no collaboration for using Zora and that they don’t experience support from colleagues. They did not know that they were allowed to choose to work with Zora (it was not mandatory). Two thirds of the professionals experienced more fun at work due to the fact that they were able to work with Zora. Almost all professionals (79%) indicated that they were happy when they worked with Zora. Most professionals felt that they received enough time to learn how to work with Zora. The support given was sufficient and aligned with their needs to use Zora. Almost every professional believed that clients are content when Zora is used; that is why they believe it is good to use Zora.

#### 3.2.2. Facilitators and Barriers Mentioned

The following were seen as facilitators: the project leader’s meetings, doing the project together, instruction training given by Zorabots and the availability of their Helpdesk by phone or email. Project leaders’ meetings are planned for all persons from in the care organizations who use Zora, to receive support and to exchange knowledge and experiences. The Helpdesk responded, in general, within two days. In some care organizations, the Wi-Fi connection was not sufficient and was considered as a barrier. An optimal connection is necessary to use Zora properly and to update Zora remotely. Although Zorabots is constantly improving Zora, many new software versions were launched in a short period. In addition, starting Zora takes more time than expected by the professionals. A care professional stated, “The long start-up time is disappointing”. For them, it was frustrating when Zora failed to start immediately. The battery life of Zora was also seen as too short according to the organizations. In 2016, Zorabots still had to develop a virtual composer to make it easier to create your own activities/compositions. This may improve the exchange of compositions between the care organizations. The care organizations mentioned that Zora’s listening proficiency and speech skills were poor (speech is unintelligible, and the responses of elderly clients are misunderstood, leading to incorrect responses). A care professional said, “Zora has a tinny voice, and the language was unclear.” This is seen as a barrier: The professionals experienced many software failures with Zora; see also Table 2 for a summary of the facilitators and barriers mentioned with regards to working with Zora.

The USE questionnaire (*N* = 19) supported the facilitators mentioned, for example, the ease of learning scores were high and also, the effect of Zora on the clients gets high scores, both >5 on a scale of 7. The ease of use and satisfaction about Zora score were lower, both scored <4 on a scale of 7. Table 3 shows the scores for 2016 and 2017; the scores of 2016 are based on a more than three months period of use.

#### 3.2.3. Professionals’ Views on the Added Value for Clients

The professionals’ view is that Zora stimulates some clients, leading to spontaneous participation. According to these professionals, Zora also has a positive effect on clients and it is highly valued. With some clients who were agitated or withdrawn, the use of Zora in a one-to-one situation gave positive results in the sense that a client who had not spoken for a while started to speak to Zora during an activity. For clients in day care and/ or with somatic problems, Zora lost credibility when having technical malfunctions. This group is more aware than residents with psychogeriatric problems that the robot is an instrument. At the same time, Zora may have added value to this group, in rehabilitation, for example.

#### 3.2.4. Practical and Implementation Questions

In spring 2017, students visited most of the care organizations to retrieve information about the implementation issues. Issues raised by the (care) professionals were about software updates and QR codes (Quick Response codes). For most of the organizations, it is hard to update the Zora software. Running software updates is important because Zorabots is continuously improving the robot. In 2017, Zorabots introduced control via QR codes. It became clear that Zora had some issues with reading the codes. Printing the QR codes on different kinds of paper or with another format solved the problems to a large extent. Students made folders on the composer containing the QR codes of all the programs in order to make it easier for care professionals to use the QR codes. 

In 2017, Zora was used more often than in 2016. In this experimental phase, the use of Zora went from ad hoc to a more structural use in 2017. Most of the organizations (*N* = 13) used Zora once or twice a week. Almost all users of Zora used the robot for movement activities while Zora was often used for cognitive training and music in combination with singing in groups sessions. 

The work experience of the professionals in relation to Zora is similar to that of 2016. The findings from the USE questionnaires revealed that the professionals still experience more fun at work and that they are contented when they work with the robot. Furthermore, professionals’ opinions are that clients are content when Zora is used. One professional said, “Clients do enjoy it when Zora is used.”

As can be seen in Table 3, the topics of the USE questionnaire in 2017 scored a bit lower than in 2016, while professionals, for example, indicated in interviews that the ease of use increased due to the introduction of the QR codes for the control of Zora.

#### 3.2.5. Facilitators and Failures Mentioned after One Year Use of Zora

The appearance of Zora has a positive effect on the clients. According to the professionals, the positive effect Zora has on clients is one of the success factors of the care robot, especially with the activities of dance, singing and games. The control pad, added by Zorabots, with the QR codes added, increased the ease of use of Zora. It makes it easier for professionals to work with Zora.

In 2017, Zorabots developed a virtual composer, as requested by the care organizations in 2016, to enhance the usability. The project leaders of the different care organizations believed that there could be other possibilities for the use of the virtual composer, namely to improve the exchange of compositions between the different care organizations in the project.

The most frequently mentioned (≥5) problems after one year of using were care professionals indicated that the comprehensibility was poor (users do not always understand the speech of the robot), the reliability (sometimes the robot is not working as expected), the listening proficiency is poor (the voice of the clients is not always audible for the robot), the starting time is still too long, the usability improved but not according to the expectations of care professionals and the stability is low (the robot falls unexpectedly when moving). A quote from one of the professionals: “Our clients have better hearing than Zora.”

Problems that have been (mostly) solved by Zorabots or by care professionals since 2016 were the network connection (Wi-Fi) improved, a virtual composer was developed that enhanced the usability, fewer software failures were experienced due to several software updates, more preprogrammed activities were available via the new software and the battery life was prolonged.

### 3.3. Clients

#### 3.3.1. Observations of the Activities

In the research period, 39 activities were observed with 245 clients. Figure 6 shows the mood of six clients, and Figure 7 shows the time on the involvement of six clients of a psychogeriatric ward. It is clear that not all clients were engaged during the ZORA activities. The involvement status of client 1 prior to, during and after the activity with Zora stayed the same, while the others were more involved, indicating a positive influence of Zora. In Figure 6, client 2 becomes more content when the activity with Zora starts, and after the activity, the mood score becomes lower. Figure 6 and Figure 7 are the results of one observation during the same activity.

**Figure 8:**
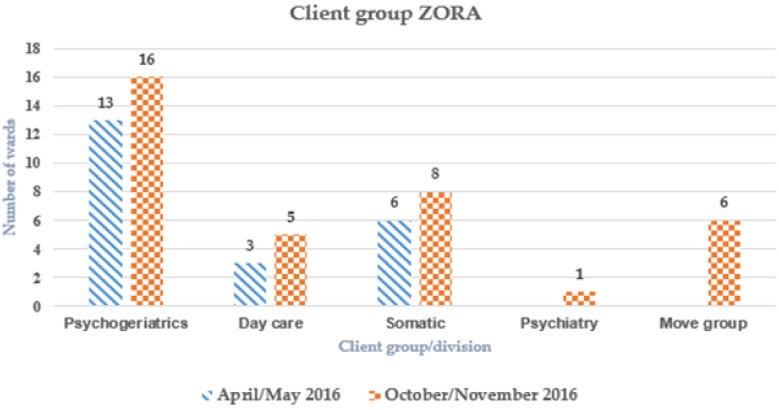
The client groups using Zora given per type of ward (2016): The “Move group” comprises clients who can do exercises to music.

**Figure 9:**
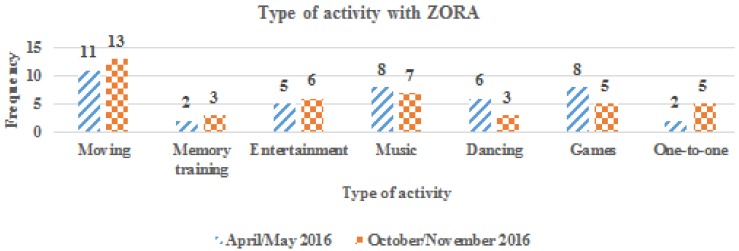
The number of locations where the different types of activities with Zora were given (2016).

From 2016 onward, a slight increase was seen in the numbers of locations that worked with Zora, and more client groups were involved. More than 15 locations can be seen because Zora can be used on more than one ward (Figure 8). Group activities to stimulate the physical activities of sedentary older adults ranged from singing together to playing old traditional games (Figure 9). Zora is also used in a one-to-one setting.

#### 3.3.2. Using Zora in Care Organizations

One of the goals after one year of use was to use Zora in more and different types of client groups. An estimate was made of the number of wards and locations based on 13 organizations. After one year of the introduction of Zora, Zora is used in approximately 59 locations/departments, so 37% of the total of 160 locations.

Zora is used in five different types of client groups, namely psychogeriatrics, day care, somatics, psychiatry and move groups. Move groups include groups with clients doing exercises to music. This is different from the music group in which music used is entertainment for listening to music and/or singing. All the locations examined used Zora for people with dementia (see Figure 8).

Zora is often used in group settings, for moving (rehabilitation), memory training (quiz), entertainment (stories), music (singing together), dancing (demo dances) or games (bingo). However, it is also possible to use the robot in a one-to-one setting. A quote about the one-to-one setting: “Especially in the one-to-one situation, Zora definitely gives added value for the clients.” Professionals used this possibility to provoke interactions and emotions and to stimulate clients. Zora was also used occasionally in the case of restlessness. Eleven of the thirteen organizations used Zora in a one-to-one situation. This was not easy to establish for the care professionals due to the fact that it’s time-intensive and that they have to type the words to communicate through Zora, but they mentioned that meaningful moments can be created, especially when using Zora for clients with dementia.

## 4. Discussion

The Zora project is in a pioneer phase in terms of the pyramid of technology innovation [11]. Looking at the pyramid of technology innovation, the use of Zora moved between the envisioned phase and the applied phase. The iterations between the operational phase and the applied phase are relevant in order to move towards a phase of acceptance. The acceptance of a new technology will occur when this innovation has reached full utilization. Full utilization not only addresses the use of the technology as instructed but also means that professionals a) are aware of the purpose of the innovation, b) are trained and competent to use the technology and c) are able to use the technology aligned with the context of the client situation and d) that the costs do not exceed the costs of normal, standard care. More focus on training could help the process toward full utilization. Zorabots advises users to follow a Zora training before using the robot, but this was not always the case. However, two project leader meetings were used to practise and to share experiences with each other, aligned with the “train the trainer” principle. Most of the end users who completed the questionnaire (*N* = 18) had training, but still, five professionals worked with Zora without any training at all.

Also, a more methodical approach by using the Normalization Process Theory of May and Finch (2009) could be used. This theory focuses on the implementation and evaluation of a complex intervention, new theories and business processes in healthcare [15]. The theory describes that it is important to focus on what people should do, not on the attitude. To reach the methodical approach, it is probably necessary to focus on collaboration and exchange but also on training, for example, by using the “train the trainer” principle. Furthermore, the iterations made the staff and management realize that they are part of the further development of the technology innovation and that they all act as co-designers of the Zora software. This means that the board and managers have to be careful in managing the expectations of the use of Zora in daily practice. The Management and Board became aware of the fact that the project with the care robot is a development project; this is apparent from the fact that almost every organization continued the project with Zora in 2017 without additional financial support given (subsidies).

While participating in the project, professionals realized that things can work out differently from what was than expected. Therefore, they needed to monitor the use of Zora carefully. During the spring of 2016, professionals experienced more difficulties than in autumn. They had negative experiences because of technical and software related issues; in autumn, they had enough experience to start with Zora. The spring and autumn trainings in 2016 were highly appreciated, as was the software update in summer. Professionals were less aware of the fact that a group activity with Zora has to be planned by themselves and that time to start with an activity has to be set aside. The findings in this study are comparable with the findings in a Finnish study about the impact of the Robot in Finnish Elderly Care [16]. They concluded that clients’ reactions differ, and care professionals should know the clients well to anticipate their reaction. The Finnish study also concluded that care professionals need time to familiarize themselves and to experiment with Zora. Furthermore, they concluded that clients are positive about Zora. However, in our study, the Zora robot did not engage all clients as can be seen in Figure 7.

The relative decrease in scores of the USE questionnaire when comparing 2017 with 2016 can be explained by the fact that the care professionals stated that improvements of Zora take longer than they expected. They see the potential, but time after time, the problems frustrates them. This is probably the reason why they are less positive about Zora in 2017. Some of the quotes are “When Zora works correctly, it is a nice tool to use.”, “Zora may contribute to the quality of care if it becomes more reliable.” and “I feel that we are not much further with Zora than a year ago.”

Prior to the visit of the organizations in 2016, all the care organizations were requested to provide their internal project proposal for using Zora at their own location. Each organization wrote a proposal about how they intend to use Zora, including a description of their project organizations, planning and strategy. The proposals of all the organizations were requested in order to examine whether their objectives are feasible. Unfortunately, not all the organizations provided their internal proposal. This could point to a lack of vision or interest in the implementation of Zora for daily practice on the wards.

In case of another social robot namely the KASPAR robot, used for children with autism spectrum disorder, professionals indicated that on an organizational or management level, vision needs to be developed and deployed on how to implement and use the KASPAR robot [17]. This indicates the relevancy of having a solid project proposal which should be authorized by the Management and Board. Moreover, in that particular study, training was only limited to instructions about the technological components and did not incorporate a social interaction (developing skills and feelings) component as recommended by Huijnen [17].

In our study, professionals had to explore which client group activities Zora can be used for optimally and what the optimal conditions are. At the end of this project, Zora was used successful in one-to-one situations and for movement activities during rehabilitation therapy. The monitoring in 2017 confirmed that the use of Zora is most successful in psychogeriatric departments. From May to November 2016, professionals gained more experience in composing a program for an activity using the preprogrammed activities available on the Zora composer. Professionals became more experienced in scheduling activities because they took into account the necessary preparation time for an activity with Zora. The professionals indicated that the number of preprogrammed activities should be higher, as they felt that composing a group activity themselves was too complicated. The online composer launched in 2017 could probably help professionals compose more specific programs because then, there is an opportunity to conduct activities without a connection with Zora and there is an option to share the conducted programs with all project leaders.

To gain more knowledge about which client groups the Zora care robot could be deployed effectively for, a more systematic way of implementation and evaluation of Zora activities is required. This current study could not answer the question “Does the Zora robot have an influence in the care organization?” because the use of Zora in the organizations was limited to one or two locations in each care organization.

In the elderly care setting, Zora has been welcomed and the clients appreciate Zora [18], but Zora is also used in other settings, for example for children with (physical or mental) disabilities. The research of Van den Heuvel, Lexis and De Witte (2017) concludes that the deployment of Zora seems to be promising in situations where clients need to learn movements again and also promising with (cognitive skills) communication and social interaction [19]. The Não humanoid robot, with software other than Zora, also has a positive influence on children with autism; it contributes to their involvement and achievement of goals in school activities [7].

### 4.1. Limitations

The quantitative data from the observations in spring and autumn of 2016 were not comparable because observations were not scored by the students in a comparable manner. In spring, students observed one activity at each location, and in autumn, two activities were observed. While in spring, each individual student observed a certain number of clients, in autumn, some of the students observed all the clients. Unfortunately, the observation method was not carried out consistently in accordance with the instructions. This is inherent in applied research in a practice-based setting when working with students. Different remarks were given in the observation forms as to how the students had experienced the observation. The following statements were made:

- “I was able to observe well, had a good view of the clients and was not distracted.”,
- “My presence did not affect clients.”,
- “My presence influenced clients; they looked at me all the time.”,
- “I participated in the activity to motivate clients to participate.” and
- “I was able to observe, but there was some distraction by people passing by.”

Another limitation was that each organization was able to set up its own project plan. The result was that some of the organizations gave professionals time allocated to Zora activities whilst others did not. This is one of the characteristics of a field trial with multiple cases. In future studies, the monitoring and evaluation should be executed in a more controlled situation. That would result in a more comprehensible comparison of the use of the Zora care robot in daily practice.

The willingness of the care organizations was very important in this research to get the information needed. Sometimes organizations were less willing; this affected the results.

### 4.2. Practical Implications

Working with a social robot like Zora is still in an experimental phase. Professionals have to be aware that things will not work perfectly immediately. Communication with staff and clients about this is crucial. Participating in such a project means acting as codesigners to enhance the technology innovation performance. In this phase, the Zora care robot showed added value when used in a rehabilitation setting to stimulate the movements of the clients while having fun. At the same, clients with psychogeriatric disorders might also benefit from the Zora activities, especially those where the Zora robot is used in one-to-one situations.

The students suggested some possible improvements based on their experiences and background. The suggestions they gave are practical and technical in nature. These suggestions have not been examined further.

- Think about how Zora can be used in the future for other activities.
- Use the function “word spotting” so Zora may respond (“listen”) better.
- Think about other ways of charging Zora, for example, with contactless charging; organizations will be able to use Zora for longer periods, for example, when they use Zora on a sheet with charging sensors, the robot can be charged while Zora is dancing.
- Use Artificial Intelligence Markup Language (AIML) to make it easier to have conversations. AIML works with programming patterns/scripts.

## 5. Conclusions

According to the professionals, the Zora robot can have a positive influence on the clients and staff. All the organizations see the potential of Zora and see possibilities for alternative ways of applying Zora in daily practice. All the care organizations are still willing to continue using robot Zora so as to offer clients alternative ways for pleasure and entertainment and rehabilitation sessions.

The results of this study are based on a single field trial, and therefore, any generalization should be treated with caution.

## Figures and Tables

**Table 1:** An overview of the professionals’ characteristics in the two research years.

Year	Age (Year) (Range (Mean))	Work Experience in Care (Years) (Range (Mean))	Gender	Received Training in Use Zora? (Yes/No)
**2016 (*N* = 44)**	16–57 (36)	0–30 (11)	77% female	35 Yes; 9 No
**2017 (*N* = 18)**	20–62 (43)	1–43 (16)	83% female	13 Yes; 5 No

**Table 2:** A summary of the facilitators and barriers regarding using Zora in 2016.

Facilitators Mentioned	Barriers Mentioned
Project leader’s meetings Doing the project together/participation in the association of care-organizations in the Utrecht region (IVVU) project (peer support) Instruction training by Zorabots Availability of the helpdesk (phone/email) Clients liked Zora’s activities. Zora stimulates people to move. Zora provides reactions from clients. Preprogrammed dances and games are funny for the clients, and clients are actively involved. Preprogrammed music makes residents reminisce.	Wi-Fi connection Too complicated to program activities on Zora Software updates of Zora composer Start-up time Battery life Missing the virtual composer (a way to make programs without a connection with Zora) Speech intelligibility and the interpretation by Zora of responses Software failures (at the start of the project) Experiencing time pressure Few preprogrammed activities available Communicating through Zora is difficult because you have to type the words on the composer at the same time (when you want to have a smooth conversation, you have to type very fast and without errors.).

**Table 3:** The scores of the modified Usefulness, Satisfaction, Ease of Use (USE) questionnaire, scored on a seven point Likert scale (except work experience five point scale). Given is the Mean (M) and Standard Deviation (SD).

Scores of the Modified USE Questionnaire	2016 (*N* = 19)	2017 (*N* = 18)
Usefulness (M ± SD)	2.94 ± 0.99	2.46 ± 0.94
Ease of use (M ± SD)	3.58 ± 1.00	3.02 ± 1.17
Ease of learning (M ± SD)	4.99 ± 1.04	4.81 ± 1.15
Satisfaction (M ± SD)	3.89 ± 1.51	3.04 ± 1.38
Effect of Zora on clients (M ± SD)	4.54 ± 1.31	3.71 ± 1.29
Work experience (M ± SD)	2.90 ± 0.38	2.89 ± 0.28

## Supplementary Materials

The following are available online at https://www.mdpi.com/2227-9032/7/1/31/s1, Supplement A: Observation scheme: Mood and Involvement. This scheme is used during the observations at the care organizations.
