# Supplementary material for: Two-Year Use of Care Robot Zora in Dutch Nursing Homes: An Evaluation Study
Source: Healthcare (Basel). 2019 Feb 19;7(1):31. doi: 10.3390/healthcare7010031 (PMC6473570; doi:10.3390/healthcare7010031)
Supplement: Supplementary file 1 [file healthcare-07-00031-s001.pdf]

Supplement A: Observation scheme: Mood and Involvement

Date:	Observer 1:<br><br>Observer 2:
Care organization:	Start time observation:
Department:	End time observation:
Sketch of the situation:	Number of the client:

**Before**	Mood (score)	Involvement (score)	Event (explain ation below)	Setting and context
15 min. vóór				Place of the activity:<br>Number of clients:<br>Number of professionals:<br>Who take care of the activity:<br>Is he/she known tot he client: ☐ ja ☐ nee<br>Explanation:……………………………………<br>Position (function) of professional(s):
10 min. vóór				
5 min. Vóór				
0 min. vóór				

Notes

Below you can give notes for extra information. Please answer the questions and write down every relevant detail. If necessary, give an explanation to make clear what you mean.

- Is Zora already in the room?
- Do the professional already know what he/she want to do with Zora?
- Is Zora ready to use?
- Are there other activities in the room?
- Is it an open space room?

Observation Mood and Involvement

**Initiation**	Setting and context
Number of the client:	Place of the activity:<br>Number of clients:<br>Number of professionals:<br>Who take care of the activity:<br>Is he/she known tot he client: ☐ ja ☐ nee<br>Explanation:……………………………………<br>Position (function) of professional(s):
Notes<br>*Below you can give notes for extra information. Please answer the questions and write down every relevant detail. If necessary, give an explanation to make clear what you mean.*<br>*- Was it hard to get the clients attention to the activity with Zora?*<br>*- How did the professional chose an activity?*	

Observation Mood and Involvement

<table>
<tr><td colspan="4">Start time activity:</td><td colspan="2">Kind of activity:</td></tr>
<tr><td>During activity with Zora</td><td>Mood (score)</td><td>Involvement (score)</td><td>Event (explanation below)</td><td>Setting and context</td><td>Number of client:</td></tr>
<tr><td>5 min.</td><td></td><td></td><td></td><td colspan="2" rowspan="12">Place of the activity:
Number of clients:
Number of professionals:
Who take care of the activity:
Is he/she known tot he client: ☐ ja ☐ nee
Explanation:…………………………………………
Position (function) of professional(s):</td></tr>
<tr><td>10 min.</td><td></td><td></td><td></td></tr>
<tr><td>15 min.</td><td></td><td></td><td></td></tr>
<tr><td>20 min.</td><td></td><td></td><td></td></tr>
<tr><td>25 min.</td><td></td><td></td><td></td></tr>
<tr><td>30 min.</td><td></td><td></td><td></td></tr>
<tr><td>35 min.</td><td></td><td></td><td></td></tr>
<tr><td>40 min.</td><td></td><td></td><td></td></tr>
<tr><td>45 min.</td><td></td><td></td><td></td></tr>
<tr><td>50 min.</td><td></td><td></td><td></td></tr>
<tr><td>55 min.</td><td></td><td></td><td></td></tr>
<tr><td>60 min.</td><td></td><td></td><td></td></tr>
</table>

Notes

Below you can give notes for extra information. Please answer the questions and write down every relevant detail. If necessary, give an explanation to make clear what you mean.

- Write down which activities with Zora be done?
- Is the one who control Zora in the same room?
- Is Zora only used? Or is something happening next to Zora?
- Worden de bewoners meegenomen in de activiteit?
- Hoe reageerde de bewoner op de activiteit?
- Vroeg de bewoner om hulp of aandacht voor de activiteit?
- Wat was de positie van ZORA tijdens de activiteit? (Op tafel, de grond, bij de bewoner op schoot?)
- Ontstaat er interactie tussen de bewoners, of tussen de bewoner en medewerker?
- Voor welke activiteit wordt ZORA op dit moment ingezet?
- Op welke manier wordt ZORA ingezet?
- Is het volledige programma gecomposed of worden alleen de door QBMT voorgeprogrammeerde activiteiten gebruikt?

Are the residents included in the activity?
- How did the resident respond to the activity?
- Did the resident ask for help or attention for the activity?
- What was the position of ZORA during the activity? (On the table, the ground, with the resident on your lap?)
- Does interaction arise between the residents, or between the resident and employee?
- For which activity is ZORA used at the moment?
- In what way is ZORA used?
- Is the complete program compiled or are only the pre-programmed activities used by QBMT used?

Observation Mood and Involvement

<table>
<tr><td>After activity</td><td>Mood (score)</td><td>Involvement (score)</td><td>Event (explanation below)</td><td>Setting en context</td><td>Number of client:</td></tr>
<tr><td>5 min.</td><td></td><td></td><td></td><td colspan="2" rowspan="3">Place of the activity:
Number of clients:
Number of professionals:
Who take care of the activity:
Is he/she known tot he client: ☐ ja ☐ nee
Explanation:…………………………………………
Position (function) of professional(s):</td></tr>
<tr><td>10 min.</td><td></td><td></td><td></td></tr>
<tr><td>15 min.</td><td></td><td></td><td></td></tr>
<tr><td colspan="6">Notes
- What happens after the activity?
- How do the residents leave?</td></tr>
</table>

Form questions for clients/residents (if possible)

Number client:

**What did you think of the activity?**	
**What did you like about the activity? What did you dislike about the activity?**	
**Would you like to do the activity again later?**	
**What kind of rating would you give the activity?**	

Form questions for employee *(if possible)*

Questions for the employee

**Find out with the employee (s) whether the ZORA activity had been planned? If so, has the activity been as planned? Why, why not?**	
**Find out with the employee (s) why was chosen for the activities and programs?**	
**Have they done the activity before?**<br>**How this was compared to other times?**	
**How did they feel that it was going?**	

Questions about the employee

**How was the healthcare professional / employee working during the activity? Was this occupied with residents or ZORA or was there room to give extra attention?** Describe the role of the employee during the activity.	

Form questions for researchers

**What went well and what was not good at observing?**	
**How was your own role in observing?**	

**Tips for the next time**	

Space for extra notes

Number client:	Care organization:

Form explanation of the scores

**Scores for mood**		
	**Mood**	Explanation
+5	Very happy / excited	Laughter, very exuberant, together with relaxed facial expression and relaxed body posture.
+3	Happy / satisfied	Laugh, hum, chuckle along with relaxed body posture
+1	Neutral	There are no clear signs of well-being or not being well-being. Person is calm and calm.
-1	Small signals of negative mood	Makes unhappy, anxious or angry impression, sombre appearance.
-3	Proper signs of negative mood	Sigh, moan, grimace, cry, scream, shout, tense posture
-5	Very sad / very negative mood	Also: very angry or very bad

**Scores for involvement**		
	**Involvement**	Toelichting
+5	Very engrossed / involved	Extremely concentrated. Do not be distracted.
+3	Properly involved	Is concentrated (with the activity), or focuses on activity or person. Do not be distracted easily.
+1	Neutral	Look at the activity with Zora, but is not actively involved, or the person is awake and alert and looks at what is happening around him, but does not actively participate in it.
-1	Withdrawn	The person is turned in on himself, is totally uninvolved in his environment. Makes an absent impression, sits and stares to the ground, stares into the distance without any signs of focus or involvement, sits with closed eyes, opens eyes occasionally.
